# Relationship between Endometrial Thickness and *In Vitro* Fertilization-Intracytoplasmic Sperm Injection Outcome

**Published:** 2013-03-06

**Authors:** Turgut Aydin, Mustafa Kara, Turktekin Nurettin

**Affiliations:** 1 Acibadem *In Vitro* Fertilization Center, Kayseri, Turkey; 2 Department of Obstetrics and Gynecology, Bozok University Medical Faculty, Yozgat, Turkey; 3 Kayseri *In Vitro* Fertilization Center, Kayseri, Turkey

**Keywords:** Endometrial Thickness, IVF-ICSI, Pregnancy Rate

## Abstract

**Background:**

This study assessed the relationship between endometrial thickness on day of human chorionic gonadotropin (hCG) administration and *in vitro* fertilizationintracytoplasmic sperm injection (IVF-ICSI).

**Materials and Methods:**

This prospective cross-sectional study included a total of 593 women. Patients were treated with either the agonist or antagonist protocol according to the clinician’s and patient’s preference. Endometrial thickness on the hCG day was measured by transvaginal ultrasonography (TV-USG). Patients were divided into four groups according to endometrial lines, as follows: <7 mm (group 1), 7-10 mm (group 2), 10-14 mm (group 3), and >14 mm (group 4).

**Results:**

Implantation rate (IR), clinical pregnancy rate (CPR), and ongoing pregnancy rate (OPR) were significantly lower in group 1 than the other three groups (p<0.05). However, there was no significant difference among groups 2, 3 and 4. Although the endometrial line in the agonist protocol was higher than in the antagonist protocol, the difference was not statistically significant.

**Conclusion:**

The chance of pregnancy appears to be lower in individuals with endometrial thickness less than 7 mm compared with those of higher value.

## Introduction

*In vitro* fertilization-intracytoplasmic sperm injection (IVF-ICSI) has been frequently performed world wide for more than two decades and many factors contribute to treatment success. Implantation is necessary for a successful pregnancy and endometrial receptivity is an important component (1). Endometrial thickness has been accepted as an indicator for endometrial receptivity and assessment of the endometrium by transvaginal ultrasonography (TVUSG) is very popular. Although endometrial receptivity is important in achieving a clinical pregnancy the studies that have intended to prove the relationship between endometrial thickness and IVF-ICSI outcome have shown conflicting results. Some authors have reported no association between endometrial thickness and pregnancy (2, 3). Some studies have shown a significant relationship between pregnancy rates and endometrial thickness (4-6), while others have reported controversial results (7, 8). In addition, there is no consensus about the cut-off value of the endometrial line that predicts treatment outcome. It would be useful to have an endometrial line cut-off value to predict the success of the IVF-ICSI treatment. The aim of this study is to assess the association between endometrial thickness on the human chorionic gonadotropin (hCG) day and IVF-ICSI outcome.

## Materials and Methods

This was a prospective cross-sectional study. The study protocol was reviewed and approved by the Ethical Committee of Medical Faculty of Bozok University. Patients provided informed consent to participate. We used two protocols, agonist (n=135) or antagonist (n=458) as previously described (3, 9). These protocols were administered according to the clinician’s choice and the patient’s preference. Endometrial line was measured by TV- USG in the midsagittal plane on the hCG day. All women were divided into four groups according to endometrial thickness. In group 1 (n=14) the endometrial line was <7 mm. In group 2 (n=177) the endometrial thickness was between 7 mm and 10 mm. In group 3 (n=366) the endometrial line was between 10 mm and 14 mm and in group 4 (n=36), the endometrial thickness was more than 14 mm.

Follicular development was monitored and dose adjusment performed according to the E_2_ level and ultrasonographic measurements. The endometrial thickness was measured by the same clinician utilizing TVUSG. When 1 or 2 follicles reached 17 mm in size, hCG (Pregnyl® 5000 IU×2, Schering-Plough, USA) was administered for final maturation. TV- USGguided needle aspiration of the follicular fluid was carried out 35 to 36 hours after hCG administration. ICSI was performed in all cases. Cleavage stage embryos were transferred into the uterine cavity on day 3 or 5. A maximum of two embryos were transferred under transabdominal ultrasound guidance. Luteal phase was supported by administering transvaginal progesterone (Crinone 8% Vaginal Gel®, Merck-Serono, Switzerland) on the oocyte pick-up day and continued for 12 days (until the serum pregnancy test). Clinical pregnancy was confirmed by the presence of a fetal sac or fetal cardiac activity at ultrasound examination two weeks after the pregnancy test.

Statistical analyses were performed using the Statistical Package for the Social Sciences (version 17.00, SPSS Inc., Chicago, IL). Data normality was assessed with the Kolmogorov-Smirnov test. Data were compared by nonparametric analysis and statistical significance was determined by the Kruskal-Wallis test. Statistical comparisons between groups were performed using the Mann-Whitney U and chi square tests. A p value <0.05 was considered significant.

## Results

Patient characteristics such as basal hormone levels, duration of infertility, body mass index (BMI), antral follicle count (AFC) and age were analyzed. The groups were homogeneous in terms of these parameters. We excluded cases in which testicular sperm extraction (TESE) procedures were performed. Also patients, whose BMI was >30, were excluded. All patients underwent standard IVF-ICSI procedures. One cycle was used for each patient.

**Fig 1:**
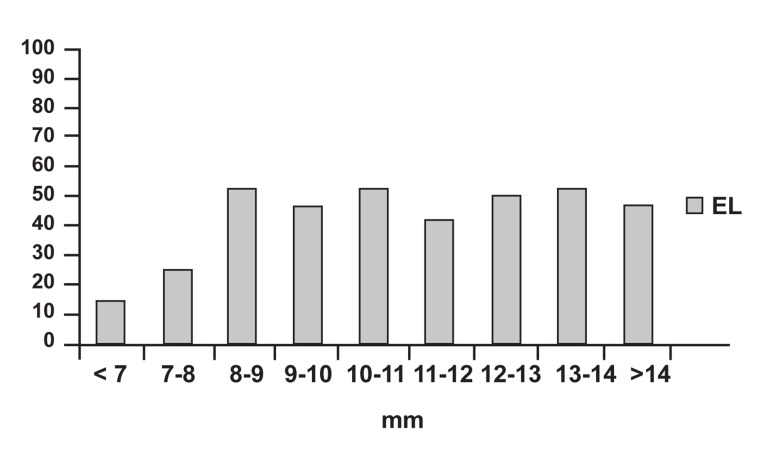
Clinical pregnancy rates according to the endometrial line. EL; endometrial line.

A total of 593 women whose ages ranged from 20 to 39 years were included in the analysis. The patient characteristics are shown in table 1. Patients’ age, duration of infertility, basal FSH levels, basal E_2_ levels, BMI, and AFC were compared but the differences were not statistically significant. The endometrial thickness ranged from 6.1 mm to 21.4 mm. Although no threshold was observed above which a pregnancy was unlikely to occur, clinical pregnancy rate (CPR) was significantly lower in cases with an endometrial thickness below 7 mm (Fig 1).

Retrieved oocyte number, transferred embryo number, and the fertilization, cleavage, and implantation rates (IR) were similar in all four groups. Implantation rate, CPR, and ongoing pregnancy rate (OPR) were significantly lower in group 1 than the other three groups (p<0.05). However, there was no significant difference among groups 2, 3 and 4 (Table 2). Endometrial thickness was lower in patients who underwent the antagonist protocol compared to the agonist protocol, however this difference was not statistically significant (Table 3).

**Table 1:** Distribution of patients’ characteristics


	Group 1 (N=14)	Group 2 (N=177)	Group 3 (N=366)	Group 4 (N=36)	P value

**Age (Y)**	27 ± 4.5	25.6 ± 3.9	27.3 ± 4.8	28.6 ± 5.7	Ns
**DI (Y)**	5.7 ± 1.1	6.7 ± 1.3	5.9 ± 1.4	4.9 ± 1.1	Ns
**Bas. FSH (IU/l)**	8.7 ± 2.1	7.4 ± 1.8	7.7 ± 1.5	7.0 ± 1.1	Ns
**Bas. E_2_ (pg/ml)**	47 ± 10.5	44 ± 9.7	41 ± 8.5	50.7 ± 12.5	Ns
**AFC**	9.0 ± 5.2	7.9 ± 4.7	6.9 ± 4.3	8.0 ± 3.3	Ns
**BMI (kg/m^2^)**	24.8 ± 4.9	26.4 ± 5.4	28.4 ± 5.5	25.2 ± 6.1	Ns


Ns; Nonsignificant, DI; Duration of infertility, Bas. FSH; Basal FSH, Bas. E_2_; Basal E_2_, AFC; Antral follicle count and BMI; Body mass index. Group 1; Endometrial line <7 mm, Group 2; Endometrial line 7-10 mm, Group 3; Endometrial line 10-14 mm and Group 4; Endometrial line >14 mm.

**Table 2:** Comparison of IVF-ICSI outcomes according to endometrial thickness on hCG day


	Group 1 (N=14)	Group 2 (N=177)	Group 3 (N=366)	Group 4 (N=36)

**RON**	10.1 ± 6.6	9.4 ± 5.8	10.8 ± 7.3	11.4 ± 7.6
**TON**	1.3 ± 0.5	1.3 ± 0.6	1.2 ± 0.3	1.4 ± 0.6
**FR (%)**	64.5 (91/141)	65.6 (1105/1685)	68.2 (2541/3724)	68.0 (273/398)
**CR (%)**	60.2 (85/141)	63.0 (1063/1685)	64.0 (2384/3724)	61.0 (243/398)
**IR (%)**	11.1 (2/18)*	20.9 (82/391)*	24.3 (188/771)*	24.4 (19/78)*
**CPR (%)**	14.3 (2/14)*	45.7 (81/177)*	48.6 (178/366)*	47.2 (17/36)*
**OPR (%)**	7.1 (1/14) *	35.5 (63/177)*	43.9 (161/366)*	41.7(15/36)*


RON; Retrieved oocyte number, TEN; Transferred oocyte number, FR; Fertilization rate, CR; Cleavage rate, IR; Implantation rate, CPR; Clinical pregnancy rate and OPR; Ongoing pregnancy rate. Group 1; Endometrial line <7 mm,Group 2; Endometrial line 7-10 mm, Group 3; Endometrial line 10-14 mm and Group; Endometrial line >14 mm. Values are mean ± SD and *; P<0.05.

**Table 3:** Distribution of endometrial thickness according to stimulation protocol


	Group 1		Group 2		Group 3		Group 4	
	N (%)	EL (mm)	N (%)	EL (mm)	N (%)	EL (mm)	N (%)	EL (mm)

**AP**	0	-	38 (21.5)	9.3 ± 1.2^a^	86 (23.5)	13.1 ± 1.6^a^	11 (30.5)	17.4 ± 2.1
**AnP**	14 (100)	6.5 ± 0.6	139 (78.5)	7.8 ± 0.9^b^	280 (76.5)	11.2 ± 1.1^b^	25 (69.5)	16.3 ± 1.8


Thickness is presented as mean ± SD. EL; Endometrial line, n; Number of patients, AP; Agonist protocol and AnP; Antagonist protocol. ^a,b^; P<0.05.

## Discussion

Although measurement of endometrial thickness is commonly utilized in clinical practice during assisted reproduction treatment, there are conflicting results regarding the association between endometrial line and IVF-ICSI outcome. Al-Ghamdi et al. have analyzed 2464 cycles and reported a positive linear relationship between the endometrial thickness measured on the day of hCG injection and CPR (6). On the other hand, Bassil assessed the endometrial features by TV-USG and claimed that endometrial measurements do not provide significant prognostic information with regards to the outcome of IVF (8).

In this prospective cross-sectional study, the relationship between endometrial line and IVF-ICSI outcome was studied. Our study showed a positive correlation between endometrial thickness and CPR. To our knowledge, this study has agreed with previous studies (5, 10, 11). There is no concensus about the minimum endometrial thickness required for a successful pregnancy. Oliveira et al. have reported that there was no clinical pregnancy when the endometrial line was less than 7 mm (12). On the other hand, successful pregnancies have been reported with endometrial lines less than 7 mm (13, 14).

There were only two clinical pregnancies (14.3%) in the current study that had an endometrial line less than 7 mm, of which one was lost. In our study the thinnest endometrial stripe was 6.1 mm. When CPR was compared with each millimeter of the endometrial line we found that the pregnancy rates decreased below the 7 mm thickness level. CPR was significantly lower in group 1 than the other groups. However, the difference among groups 2, 3, and 4 were not statistically significant. Chen reported that CPR was 23.0% (12/52) in patients whose endometrial line was below 7 mm (15). These values were higher than our results. Therefore, we should perform IVF-ICSI in these patients.

Implantation is necessary for a successful pregnancy and requires a healthy endometrial receptivity (16). We have noted IRs of 11.1% (group 1), 20.9% (group 2), 24.3% (group 3), and 24.4% (group 4), which was statistically significant. These findings were consistent with CPR results. OPR was assessed and found to be 7.1% (group 1), 35.5% (group 2), 43.9% (group 3), and 41.7% (group 4), which was statistically significant.

In light of these data, the measurement of endometrial thickness on the day of hCG administration remains important. Several studies have reported that CPR increases as endometrial thickness increases (6, 10). Our results, to a point, were consistent with these studies. CPR and OPR increased as the endometrial line increased, however when the endometrial line was more than 14 mm there was no increase in pregnancy rate. These differences were not statistically significant. Endometrial thickness was compared according to the utilized protocol. The endometrial lining tended to be lower in the antagonist protocol compared to the agonist protocol, however this difference was not statistically significant.

## Conclusion

We have researched the association between endometrial thickness and IVF-ICSI outcome. Our results indicate that close monitoring of the endometrial line during IVF-ICSI treatment is recommended. Eventhough there is a lack of agreement with regards to the minimum endometrial thickness required for a successful pregnancy, our results suggest that CPR will be low when the endometrial thickness is less than 7 mm. However, large prospective and randomized trials are required to assess the predictive value of endometrial thickness measurement.
